# Perinatal risks associated with infertility and medically assisted reproduction: a population-based cohort study

**DOI:** 10.1093/hropen/hoaf020

**Published:** 2025-05-08

**Authors:** Stephanie K Y Choi, Wentao Li, Christos Venetis, William Ledger, Kei Lui, Katie Harris, Robert J Norman, Louisa R Jorm, Georgina M Chambers

**Affiliations:** National Perinatal Epidemiology and Statistics Unit (NPESU), Centre for Big Data Research in Health, Faculty of Medicine and Health, University of New South Wales (UNSW Sydney), Sydney, Australia; National Perinatal Epidemiology and Statistics Unit (NPESU), Centre for Big Data Research in Health, Faculty of Medicine and Health, University of New South Wales (UNSW Sydney), Sydney, Australia; National Perinatal Epidemiology and Statistics Unit (NPESU), Centre for Big Data Research in Health, Faculty of Medicine and Health, University of New South Wales (UNSW Sydney), Sydney, Australia; Faculty of Medicine and Health, School of Clinical Medicine, University of New South Wales, Sydney, Australia; Faculty of Medicine and Health, School of Clinical Medicine, University of New South Wales, Sydney, Australia; Faculty of Medicine and Health, The George Institute for Global Health, University of New South Wales, Sydney, Australia; The Robinson Research Institute, The University of Adelaide, Adelaide, South Australia, Australia; Centre for Big Data Research in Health, Faculty of Medicine and Health, University of New South Wales, Sydney, Australia; National Perinatal Epidemiology and Statistics Unit (NPESU), Centre for Big Data Research in Health, Faculty of Medicine and Health, University of New South Wales (UNSW Sydney), Sydney, Australia

**Keywords:** reproductive technologies, population-based studies, birth rate, parental characteristics, social inequality, Australia

## Abstract

**STUDY QUESTION:**

Are the risks of adverse perinatal outcomes in singletons born from medically assisted reproduction (MAR) mainly associated with underlying parental infertility, or are they primarily linked to the MAR treatments?

**SUMMARY ANSWER:**

While MAR-conceived singletons have increased risks of preterm birth, admission to neonatal intensive care unit (NICU), and hospital admission in early life, these risks are mainly associated with the underlying parental infertility that led to the use of MAR technologies.

**WHAT IS KNOWN ALREADY:**

Children born from MAR are at increased risk for some adverse perinatal and infant outcomes. However, to what extent this risk is associated with infertility or MAR treatment remains unclear. This knowledge gap arises from the challenge in disentangling the effects of infertility and MAR treatment, given that parental infertility necessitates the use of MAR treatment.

**STUDY DESIGN, SIZE, DURATION:**

This is a statewide longitudinally data-linked population-based cohort study conducted in New South Wales, Australia, involving all singleton infants born (liveborn or stillborn) between 2009 and 2017.

**PARTICIPANTS/MATERIALS, SETTING, METHODS:**

We applied two comparisons to isolate the associations of infertility from its treatment: (i) MAR infants versus naturally conceived infants from fertile parents (NC-fertile), and (ii) MAR infants versus naturally conceived infants from parents who had a history of infertility (NC-infertile). The study cohort consisted of 824 639 singleton infants, of whom 27 796 (3.4%) were conceived through ART and 13 574 (1.6%) through ovulation induction/intrauterine insemination (OI/IUI), while 783 269 (95.0%) of the infants were naturally conceived (747 018 NC-fertile controls and 36 251 NC-infertile controls). We used the inverse probability of treatment weighting method to make MAR infants comparable with each of the two NC control groups. We then calculated the adjusted risk differences (aRDs) in these propensity score-weighted cohorts. In the subgroup analyses of different forms of ART treatment (ICSI vs IVF and fresh vs frozen embryo transfer), we reweighted the study cohort and compared these subgroups with the two NC control groups separately.

**MAIN RESULTS AND THE ROLE OF CHANCE:**

Singletons conceived through ART had a higher risk for preterm birth (aRD 25.7 per 1000 infants, 95% CI 21.3–30.0), admission to NICU (aRD 8.4 per 1000 infants, 95% CI 1.2–15.6), and hospital admission within 2 years of life (aRD 24.6 per 1000 infants, 95% CI 17.2–32.0) compared to NC-fertile controls. These risks were notably reduced when compared to NC-infertile controls (aRD 9.5 per 1000 infants, 95% CI 4.8–14.2 for preterm birth; −0.7 per 1000 infants, 95% CI −8.0 to 6.6 for NICU admission; and 10.6 per 1000 infants, 95% CI 2.5–18.7 for hospital admission within 2 years of life). ART-conceived singletons also had a higher risk of stillbirth compared to NC-fertile controls (aRD 1.5 per 1000 infants, 95% CI 0.4–2.7), which decreased when compared to NC-infertile controls (aRD 0.8 per 1000 infants, 95% CI −0.4 to 2.1). Similar patterns were observed for OI/IUI-conceived infants.

Compared to NC-fertile controls, infants conceived by either ICSI (preterm birth: aRD 18.4 per 1000 infants, 95% CI 11.9–24.8; hospital admission: aRD 43.4 per 1000 infants, 95% CI 31.4–55.4) or IVF (preterm birth: aRD 26.4 per 1000 infants, 95% CI 18.7–34.1; hospital admission: aRD 30.2 per 1000 infants, 95% CI 17.0–43.4) had higher risks, but these risks decreased significantly when compared to NC-infertile controls (ICSI: preterm birth aRD 7.7 per 1000 infants, 95% CI 1.9–13.5; hospital admission aRD 17.0 per 1000 infants, 95% CI 6.9–27.2; IVF: preterm birth aRD 13.1 per 1000 infants, 95% CI 6.6–19.7; hospital admission aRD 0.9 per 1000 infants, 95% CI −10.3 to 12.0).

Infants conceived by fresh ART transfers had higher risks of preterm birth (aRD 33.7 per 1000 infants, 95% CI 27.6–39.9) and hospital admission (aRD 33.7 per 1000 infants, 95% CI 23.5–43.9) compared to NC-fertile controls, with reduced risks when compared to NC-infertile controls (preterm birth: aRD 20.5 per 1000 infants, 95% CI 14.1–26.9; hospital admission: aRD 17.8 per 1000 infants, 95% CI 7.3–28.3). These risks were substantially lower for those conceived by frozen embryo transfers and came close to zero when compared to NC-infertile controls. However, frozen embryo transfer increased the risks of LGA (aRD 28.5 per 1000 infants, 95% CI 20.5–36.6) compared to NC-fertile controls, and this risk persisted when compared to NC-infertile controls.

**LIMITATIONS, REASONS FOR CAUTION:**

The observational nature and use of administrative data may carry a risk of misclassification or unmeasured confounding. We only included singletons because the risk profile for multiple births differs significantly. Parents with a history of infertility who achieved natural pregnancy likely had less severe conditions, potentially underestimating the contribution of parental infertility to perinatal risks.

**WIDER IMPLICATIONS OF THE FINDINGS:**

The primary factor contributing to the increased risks of certain adverse perinatal outcomes is the underlying parental infertility that necessitates ART treatment. However, ART procedures also contribute to the risks to some extent; this study highlights the importance of careful monitoring and of reserving ART for where ART treatment is indicated.

**STUDY FUNDING/COMPETING INTEREST(S):**

This study is funded by the Australian National Health and Medical Research Council (APP1127437). The sponsors had no role in: the design and conduct of the study; collection, management, analysis, and interpretation of the data; preparation, review, or approval of the manuscript; and decision to submit the manuscript for publication. S.K.Y.C. is an employee of Sanofi, but this study was conducted before this role. W.L. declared research grant support from the Australian National Health and Medical Research Council for other projects. C.V. declared having received honoraria for invited lectures in scientific meetings/conferences, and/or having travel support, and/or being a member of advisory boards for Merck Ltd, Merck Sharpe & Dohme, Ferring, Organon, Vianex, Gedeon-Richter, and IBSA. C.V. was a minority shareholder of Virtus Health Ltd until June 2022 and a member of the Board of Directors of the Fertility Society of Australia and New Zealand and a member of the Executive Board of the ‘Doctors in ART’ of the Fertility Society of Australia and New Zealand between 2019 and 2023. C.V. currently serves ESHRE as a Senior Deputy of the Steering Committee of the Special Interest Group Reproductive Endocrinology. He is also a fertility specialist offering his services to private patients. W.L. is a minority shareholder of CHA SMG Australia. R.J.N. declared research grant support from the Australian National Health and Medical Research Council for other projects, consulting or speaking fees from VinMec Vietnam, Westmead Fertility, Flinders Fertility, and Proadwise India, payment for lectures from Cadilla Pharma, and travel support from Merck Ltd L.R.J. declared research grant support from the Australian National Health and Medical Research Council for other projects. G.M.C. declared research grant support from the Australian National Health and Medical Research Council for other projects. G.M.C. is the Director of the National Perinatal Statistics and Epidemiology and Statistics Unit, UNSW, which prepares annual reports and benchmarking reports from the Australian and New Zealand Assisted Reproductive Technology Database (ANZARD). The remaining authors have no relevant disclosures for this study.

**TRIAL REGISTRATION NUMBER:**

N/A.

WHAT DOES THIS MEAN FOR PATIENTS? Children born from medically assisted reproduction (MAR), such as IVF and other treatments, are known to face higher risks of certain health problems around birth and in their early years. However, it is unclear whether these risks are related to the treatments themselves or to the infertility that led to these treatments being sought. This study aims to clarify this question.

We studied over 800 000 single babies born in New South Wales, Australia, from 2009 to 2017. We compared the health outcomes of children conceived through MAR with two groups: children conceived naturally by parents without fertility issues, and children conceived naturally by parents who had fertility issues beforehand.

We found that children conceived through MAR had higher risks of being born early, needing intensive care, and being admitted to hospital in their first 2 years of life compared to children conceived naturally by parents without fertility issues. However, when compared to children of parents with fertility issues who conceived naturally, these risks were much lower but did not disappear completely. This suggests that most of the health risks seen in children born through MAR are related to the underlying infertility of the parents, rather than the treatments themselves. That said, the treatments can contribute a small additional risk. These findings highlight the importance of carefully monitoring MAR treatments and using them only when medically indicated.

## Introduction

Nearly 100 000 live births are attributed to ART each year in the US alone, with nearly 80% of the ART cycles using ICSI for fertilization and over 80% of embryos transferred as frozen-thawed embryos (National ART Summary | CDC, 2021). Globally, more than 2.5 million ART cycles are performed annually, resulting in over half a million deliveries and accounting for more than 5% of births in some developed countries (Fauser, 2019). Ovulation induction/intrauterine insemination (OI/IUI) is estimated to produce a similar number of births (Duwe *et al.*, 2010; Kulkarni *et al.*, 2013).

Previous studies have compared perinatal outcomes of children born via ART to those of naturally conceived children. Singletons conceived through ART are more likely to be at higher risk of adverse perinatal outcomes (Berntsen *et al.*, 2019), including preterm birth, low birth weight (Qin *et al.*, 2017), perinatal death, and stillbirth (Marino *et al.*, 2014; Berntsen *et al.*, 2019). Furthermore, there is some evidence that ART singletons are more likely to be admitted to hospital than their naturally conceived counterparts during childhood (Koivurova *et al.*, 2007). However, an enduring question for clinicians and patients is whether these higher risks are related to underlying parental infertility or its treatment (Henningsen and Pinborg, 2014; Berntsen *et al.*, 2019). This raises a further question of whether more caution is needed in the use of ART. Unfortunately, using overall naturally conceived children as a control group does not allow this question to be answered because it is not possible to separate the effect of infertility from its treatment (Berntsen *et al.*, 2019).

Debates also remain regarding the specific roles that ART variations play in influencing perinatal outcomes. Previous research highlights differences in perinatal outcomes associated with certain ART methods. ICSI singletons appear to have a lower risk of preterm birth compared to IVF singletons (Pinborg *et al.*, 2013). Frozen embryo transfers are associated with reduced risks of preterm birth and low birth weight compared to fresh transfers but carry higher risks of large-for-gestational-age (LGA) infants and macrosomia (Wennerholm *et al.*, 2013; Luke *et al.*, 2017; Maheshwari *et al.*, 2018; Westvik-Johari *et al.*, 2021). However, directly comparing ART variations could be challenging, as different treatments are often used for specific indications and populations. To address this, an understanding of the standalone effects of these treatments on adverse perinatal outcomes while controlling for infertility is essential.

To decipher whether the adverse outcomes in children conceived by MAR, including ART and OI/IUI, are associated mainly with infertility or its treatment, we established a statewide, longitudinally linked population-based cohort of singleton infants. In addition, as a secondary aim, we aimed to explore the possible differences in the effects of different forms of ART, i.e. ICSI versus IVF and fresh versus frozen embryo transfer. Uniquely, we included two control groups: infants naturally conceived by parents with and without a history of infertility. The controls whose parents had a history of infertility primarily evaluate the effect of MAR and the controls whose parents were fertile assess the joint effect of infertility and MAR.

## Materials and methods

### Study design and population

We performed a population-based longitudinal cohort study of all singleton liveborn and stillborn infants (≥400 g birthweight or ≥20 weeks’ gestation) born in New South Wales (NSW), Australia between 2009 and 2017. Details regarding the data linkage have been reported previously (Chambers *et al.*, 2021; Choi *et al.*, 2022; Venetis *et al.*, 2023). In brief, we linked birth records from the Australia and New Zealand Assisted Reproduction Database (ANZARD): a clinical quality registry that contains detailed information on all ART treatments performed in Australia and New Zealand, with administrative health data from state and national sources. These datasets provided comprehensive information encompassing birth registrations, perinatal and birth details, medical services utilized, prescribed medications, hospitalizations, and mortality records (Supplementary Table S1). The data linkage and birth concordance rates between ANZARD and state-based datasets exceeded 94% (Chambers *et al.*, 2021).

In Australia, the national health insurance scheme, Medicare, provides substantial subsidies for a wide range of MAR treatments, including ART, IUI, and OI. These subsidies cover more than two-thirds of the costs for clinically necessary ART procedures and are not subject to restrictions based on age, parity, or the number of prior cycles. Between 2009 and 2017 in NSW, the proportion of births resulting from ART increased from 5.1% to 6.7%. In contrast, the proportion of births conceived through OI and IUI remained relatively stable at ∼2% (Choi *et al.*, 2022).

We classified infants who were born as singletons based on their method of conception and the fertility history of their parents. Those who were conceived through MAR included ART-conceived and OI/IUI-conceived infants. We first identified if the birth was due to ART through the cycle information in ANZARD, service codes related to ART in the Medicare Benefits Schedule, and ART-specific fertility medicine dispensing in the Pharmaceutical Benefits Scheme. Only autologous cycles were included. Based on ANZARD data, we categorized ART-conceived infants into two comparisons: those conceived through ART-IVF versus ART-ICSI, and those resulting from ART-fresh versus ART-frozen embryo transfers. After excluding ART-conceived births, we determined if the birth was due to OI from the Pharmaceutical Benefits Scheme for the dispensing records of OI-related medicines. We then identified if the birth was due to IUI using data from ANZARD and the Medicare Benefits Schedule. Finally, births not assigned to any MAR cohort were then assumed to have been naturally conceived (NC) and were grouped based on whether there was parental infertility history (NC-infertile control group) or no evidence of previous infertility (NC-fertile control group). A history of infertility in the parents of NC-infertile infants was identified by previous fertility treatment or infertility investigations from the earliest available fertility treatment record until 1–3 months before the date of index conception (Supplementary Table S2). The history of fertility treatment was assessed using ART-specific procedures recorded in ANZARD, Pharmaceutical Benefits Scheme records for fertility-specific medicine (ART-specific or general), and Medicare Benefits Schedule records for fertility procedures. A history of infertility investigations was identified using Medicare Benefits Schedule records for fertility-related testing procedures. More details are described elsewhere (Choi *et al.*, 2022; Venetis *et al.*, 2023).

The NSW Population and Health Services Research Ethics Committee (2017/HRE1202) and the Australian Institute of Health and Welfare Ethics Committee (AIHW) (EO2017/4/420) approved the data-linkage study. We reported this study according to the Strengthening the Reporting of Observational Studies in Epidemiology (STROBE) reporting guidelines.

### Perinatal and infant outcomes

Outcomes of interest included: stillbirth (a foetal death before the delivery of a baby at 20 weeks gestation or more, and/or weighing 400 g or more; Australian Institute of Health and Welfare, 2024b), perinatal mortality (stillbirth or neonatal death, i.e. death within 28 days after live birth), preterm birth (a birth that occurs before the 37 completed weeks of pregnancy), very early preterm birth (a birth that occurs before the 32 completed week of pregnancy), small-for-gestational-age (SGA) (Dobbins *et al.*, 2012) (birth weight of less than 10th percentile for gestational age), LGA (Dobbins *et al.*, 2012) (birth weight above 90th percentile for gestational age), APGAR score at 5 min <7, admission to special care nursery/neonatal intensive care unit (SCN/NICU), hospital admissions, and infant death in the first 2 years of life.

### Maternal and paternal covariates

Details regarding how covariates were defined and identified from registries have been reported previously (Venetis *et al.*, 2023). Covariates should be associated with infertility and its treatment as well as perinatal outcomes. These include the ages of the mother and father, year of birth, parity, and whether the mother resided in a major city or socioeconomically disadvantaged area. Covariates also included whether the mother was an immigrant to Australia and smoked during pregnancy. Additionally, we included several maternal comorbidities as covariates, following established frameworks (Quan *et al.*, 2005; Pratt *et al.*, 2018). These comorbidities included cervical incompetence, fibroid uterus, congenital uterine anomalies, cardiovascular disease, asthma during pregnancy, alcohol, and drug dependence and renal disease, as well as a history of preterm birth, miscarriage, antepartum haemorrhage, and prelabour-preterm rupture of membranes. These comorbidities were assessed based on hospital admission records with a 2-year look-back period using the International Classification of Diseases code (Roberts *et al.*, 1998).

### Statistical analysis

We summarized baseline characteristics in infants according to their mode of conception and parental fertility status as number and percentage. We computed the crude incidence (%) of adverse perinatal and infant outcomes in ART and OI/IUI infants and the two NC control groups (NC-infertile and NC-fertile).

We used the stabilized inverse probability of treatment weighting method (nonparametric generalized boosted model) to make MAR-conceived infants comparable with each of the two NC control groups (Chesnaye *et al.*, 2022). We computed the propensity score for the mode of conception using maternal and paternal covariates stated above. We then used a generalized linear regression with binomial distribution and identity link in our propensity score-weighted cohort to compute adjusted risk differences (aRDs) for outcomes between the cohorts (Li *et al.*, 2013; Naimi and Whitcomb, 2020). Because some of the data involved infants of the same mother, we used cluster robust standard errors (Taylor series linearization) to control for correlation on outcomes between infants. There were three comparisons:

(i) MAR-conceived infants versus naturally conceived control infants who were born to couples with no history of infertility (NC-fertile) to estimate the joint effect of infertility and MAR treatment; (ii) MAR-conceived infants versus naturally conceived control infants who were born to couples with a history of infertility (NC-infertile) to primarily evaluate the effect of MAR treatment; and (iii) NC-infertile controls versus NC-fertile controls to estimate the effect of underlying infertility.

In the subgroup analysis of different forms of ART treatment, we reweighted the study cohort and compared these subgroups of ART with the two NC control groups using the same method as the main analysis.

All tests were two-sided, and the level of significance was 0.05. The analysis was performed using R environment version 4.03 (Vienna, Austria).

## Results

### Characteristics of the study population

**Figure 1.**
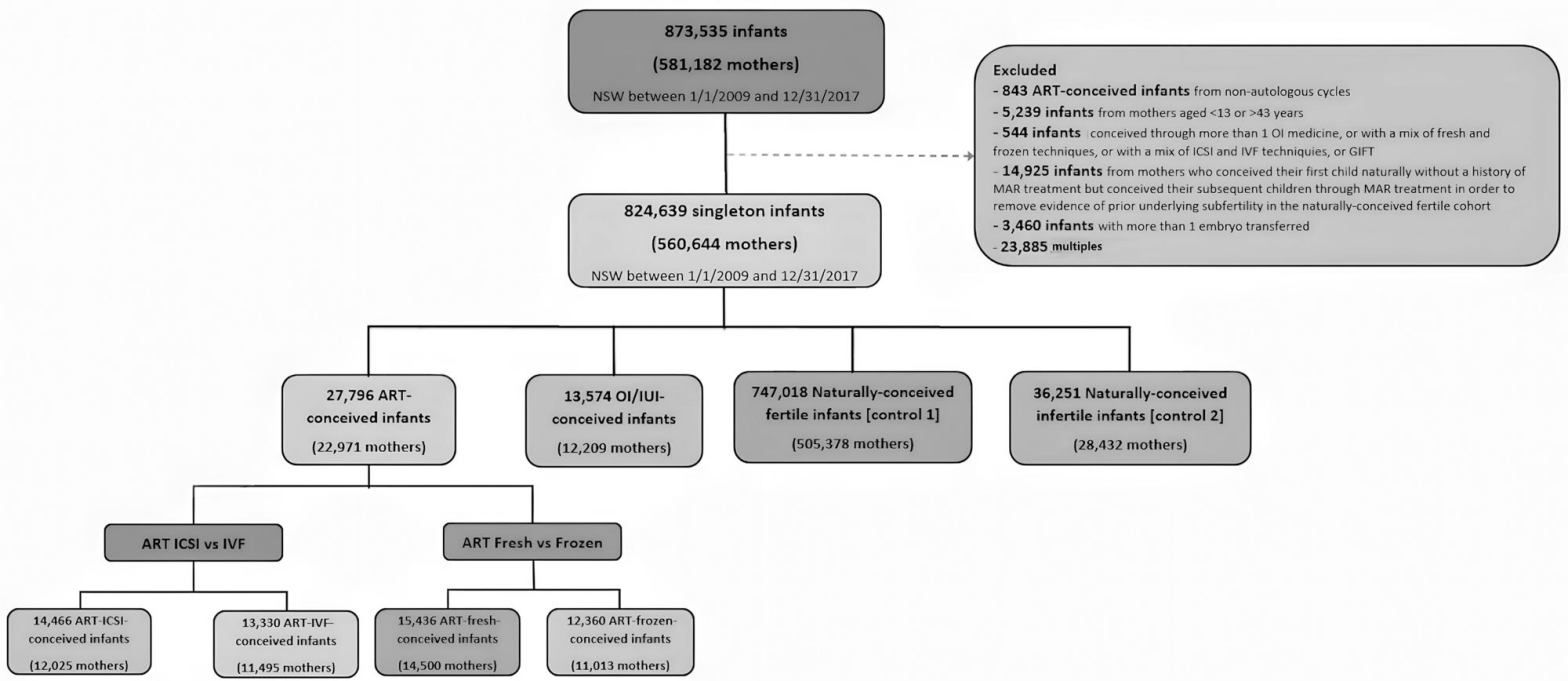
**Flowchart of cohort formation.** OI/IUI, ovulation induction and/or intrauterine insemination; NC-infertile, naturally conceived infertile births were conceived naturally by couples with a history of infertility; NC-fertile, naturally conceived fertile births were conceived naturally by couples without a history of infertility.

The study cohort consisted of 824 639 singleton infants, of whom 27 796 (3.4%) were conceived through ART, 13 574 (1.6%) were conceived through OI/IUI, while 783 269 (95.0%) of the infants were naturally conceived (747 018 NC-fertile controls and 36 251 NC-infertile controls). Among the ART-conceived infants, 13 330 (48.0%) used IVF, while 14 466 (52.0%) used ICSI as the fertilization method. Regarding the type of embryo transferred, 15 436 (55.5%) were conceived from fresh embryos, while 12 360 (44.5%) were from frozen/thawed embryos (Fig. 1).

Mothers and their partners of ART-conceived infants and NC-infertile controls were older than those who were conceived through OI/IUI or were NC-fertile controls. Smoking during pregnancy was most common in the NC-fertile controls (10.9%) and least prevalent in ART pregnancies (1.5%). Mothers who conceived using ART or OI/IUI were more likely to be nulliparous than those who had natural conception, and those who used ART to conceive were less likely to reside in socially disadvantaged areas. Pre-existing comorbidities were most prevalent in mothers of OI/IUI-conceived infants and NC-infertile controls, followed by those who had ART, with the prevalence of comorbidities lowest in the NC-fertile control (Table 1).

**Table 1.** Baseline characteristics for MAR-conceived births and naturally conceived births, Singleton, NSW, 2009–2017 (N = 824 639).

	ART-conceived (N = 27 796)	OI/IUI-conceived (N = 13 574)	NC-fertile (control 1) (N = 747 018)	NC-infertile (control 2) (N = 36 251)
**Maternal age, at delivery**				
<30 years	2887 (10.4%)	4226 (31.1%)	312 415 (41.8%)	5156 (14.2%)
30–34 years	9456 (34.0%)	5441 (40.1%)	258 539 (34.6%)	12 581 (34.7%)
35–39 years	11 229 (40.4%)	3217 (23.7%)	145 762 (19.5%)	13 589 (37.3%)
≥40 years	4224 (15.2%)	690 (5.1%)	30 302 (4.1%)	4925 (13.6%)
**Other parent’s age, at delivery**				
<30 years	1421 (5.1%)	2249 (16.6%)	183 711 (24.6%)	2442 (6.7%)
30–34 years	6672 (24.0%)	4720 (34.8%)	228 105 (30.5%)	9076 (25%)
35–39 years	9566 (34.4%)	3830 (28.2%)	172 702 (23.1%)	12 556 (34.6%)
≥40 years	8692 (31.3%)	2143 (15.8%)	101 903 (13.6%)	10 327 (28.5%)
**Baby’s sex, male**	14 764 (53.1%)	6970 (51.4%)	383 714 (51.4%)	18 598 (51.3%)
**Smoking during pregnancy**	414 (1.5%)	470 (3.5%)	81 169 (10.9%)	1714 (4.7%)
**Nulliparous**	17 856 (64.2%)	8662 (63.8%)	321 356 (43%)	10 467 (28.9%)
**Australia-born**	18 290 (65.8%)	9198 (67.8%)	482 428 (64.6%)	23 747 (65.5%)
**Residing in most socially disadvantaged areas**	8010 (28.1%)	5910 (43.5%)	345 959 (46.3%)	14 421 (39.8%)
**Residing in major cities**	24 519 (88.2%)	11 181 (82.4%)	573 440 (76.8%)	31 021 (85.6%)
**Year of birth**				
2009	2554 (9.2%)	1782 (13.1%)	83 222 (11.1%)	3858 (10.6%)
2010	2722 (9.8%)	1442 (10.6%)	82 523 (11.1%)	4064 (11.2%)
2011	2621 (9.4%)	1784 (13.1%)	83 184 (11.1%)	4037 (11.1%)
2012	2988 (10.8%)	1509 (11.1%)	85 242 (11.4%)	4052 (11.2%)
2013	3134 (11.3%)	1491 (11%)	82 589 (11.1%)	4035 (11.1%)
2014	3269 (11.8%)	1555 (11.5%)	82 886 (11.1%)	3898 (10.8%)
2015	3298 (11.9%)	1405 (10.4%)	82 133 (11%)	3973 (11%)
2016	3614 (13.0%)	1320 (9.7%)	84 050 (11.3%)	4050 (11.2%)
2017	3596 (12.9%)	1286 (9.5%)	81 189 (10.9%)	4284 (11.8%)
**Pre-existing comorbidities** ^a^				
Diabetes	1890 (6.8%)	2524 (18.6%)	14 199 (1.9%)	2899 (8.0%)
Hypertension	527 (0.9%)	188 (1.4%)	5930 (0.8%)	464 (1.3%)
Alcohol and drug dependence	47 (0.2%)	25 (0.2%)	6105 (0.8%)	81 (0.2%)
Mental disorder	2252 (8.1%)	1359 (10.7%)	80 331 (10.8%)	3988 (11.0%)
Chronic airway disease	2307 (8.3%)	1456 (10.7%)	69 319 (9.3%)	3646 (10.1%)
Cancer	105 (0.4%)	44 (0.3%)	1571 (0.2%)	161 (0.4%)
Cardiovascular disease	450 (1.6%)	290 (2.1%)	12 264 (1.6%)	977 (2.7%)
Thyroid	1570 (5.7%)	554 (4.1%)	13 373 (1.8%)	1601 (4.4%)
Gastro-oesophageal reflux disease	1871 (6.7%)	1271 (9.4%)	49 303 (6.6%)	3363 (9.3%)
Epilepsy	160 (0.6%)	140 (1.0%)	6271 (0.8%)	271 (0.8%)
Anaemia and coagulation	2384 (8.6%)	350 (2.6%)	10 371 (1.4%)	2033 (5.6%)
Inflammation/pain	4673 (16.8%)	2344 (17.3%)	104 968 (14%)	6403 (17.7%)
Steroid responsive disease	1594 (5.7%)	624 (4.6%)	28 128 (3.8%)	1836 (5.1%)
Irritable bowel disease	227 (0.8%)	93 (0.7%)	3193 (0.4%)	278 (0.8%)
Liver disease	78 (0.3%)	35 (0.3%)	2312 (0.3%)	150 (0.4%)
Rheumatic disease	1872 (6.7%)	1130 (8.3%)	47 260 (6.3%)	2992 (8.3%)
Obesity	86 (0.4%)	89 (0.7%)	2566 (0.3%)	292 (0.8%)
**Pregnancy-related comorbidities**				
Cervical incompetence	299 (1.1%)	101 (0.7%)	2341 (0.3%)	404 (1.1%)
Fibroid uterus	124 (0.5%)	29 (0.2%)	1035 (0.1%)	99 (0.3%)
Congenital uterine anomalies	28 (0.1%)	21 (0.2%)	414 (0.1%)	41 (0.1%)
Cardiovascular disease	294 (1.1%)	119 (0.9%)	5637 (0.8%)	332 (0.9%)
Asthma during pregnancy	412 (1.5%)	201 (1.5%)	9914 (1.3%)	639 (1.8%)
Alcohol and drug dependence	10 (0.04%)	7 (0.1%)	3460 (0.5%)	35 (0.1%)
Renal disease	217 (0.8%)	92 (0.7%)	4283 (0.6%)	259 (0.7%)
History of preterm births	282 (1.0%)	80 (0.6%)	8970 (1.2%)	912 (2.5%)
History of miscarriage	2312 (8.3%)	1197 (8.8%)	32 409 (4.3%)	3324 (9.2%)
History of antepartum haemorrhage	156 (0.6%)	37 (0.3%)	4480 (0.6%)	406 (1.1%)
History of prelabour-preterm rupture of membranes	549 (2.0%)	147 (1.1%)	20 464 (2.7%)	1632 (4.5%)

OI/IUI, ovulation induction and/or intrauterine insemination; MAR, medically assisted reproduction.

Naturally conceived infertile (NC-infertile) births that were conceived naturally by mothers with a history of infertility; naturally conceived fertile (NC-fertile) births that were conceived naturally by mothers without a history of infertility.

a Pre-existing comorbidities were determined 2 years before the date of conception.

Supplementary Table S3 provides characteristics of births by the fertilization method (IVF and ICSI) and type of embryo transfer (fresh and frozen). The inverse probability weighting achieved a satisfactory balance for all the covariates between MAR-conceived infants and the two NC infant cohorts. (Supplementary Figs S1 and S2).

### Risk of adverse perinatal outcomes associated with ART and OI/IUI

In the unweighted cohort, the crude prevalence of perinatal death, stillbirth, preterm birth, APGAR score at 5 min <7, admission to NICU, and hospital admission <2 years of life was higher in ART- and OI/IUI-conceived infants than in either of the two NC controls. Also, NC-infertile controls had a higher prevalence of these risks than NC-fertile controls (Supplementary Fig. S3 and Supplementary Table S4).

**Figure 2.**
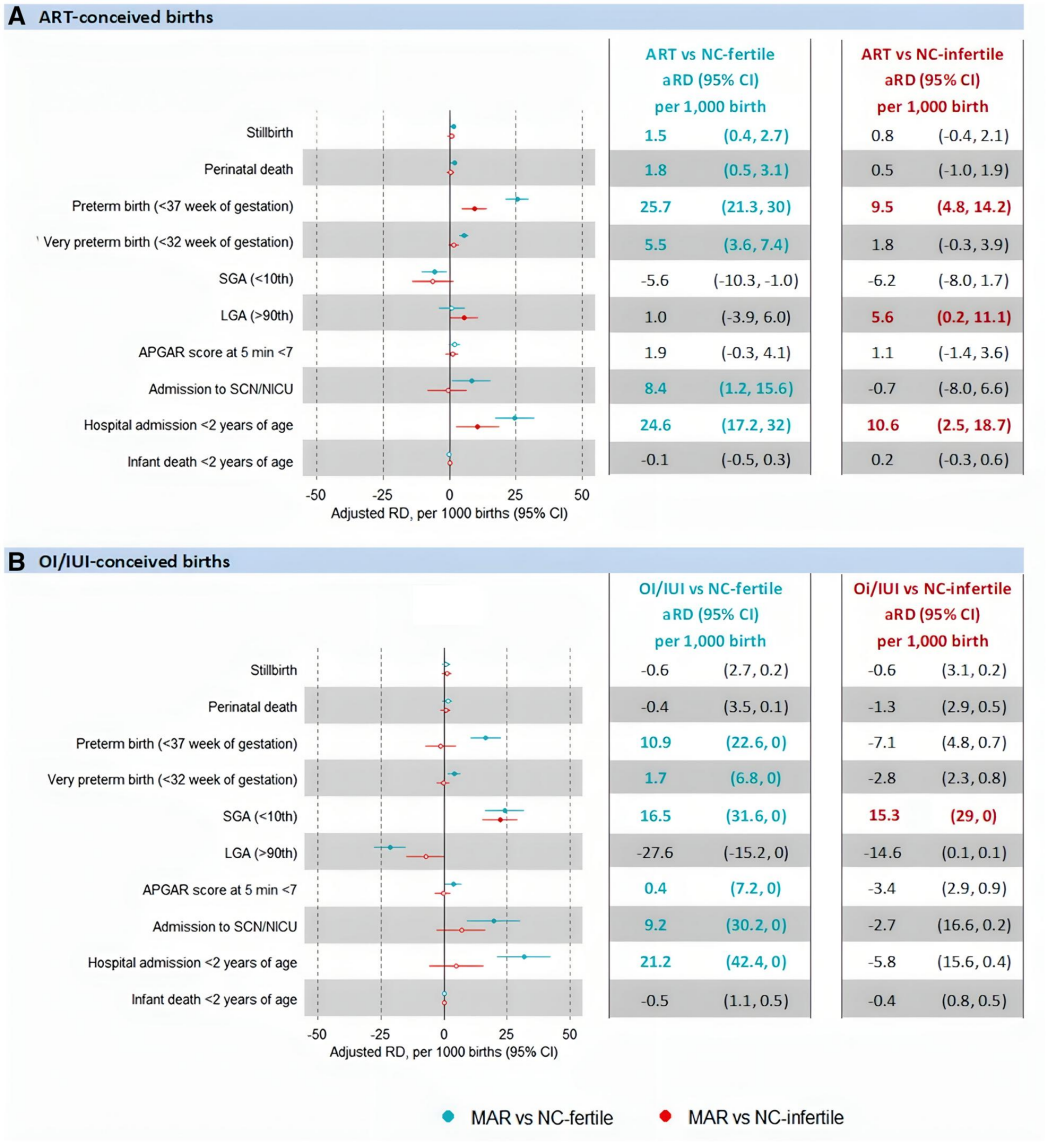
**Adjusted risk difference (aRD) for adverse perinatal outcomes for ART and OI/IUI-conceived births compared to the two groups of naturally conceived (NC) controls, Singletons, NSW, 2009–2017 (N = 824 639).**  **A**) ART-conceived births. **B**) OI/IUI-conceived births. MAR, medically assisted reproduction; OI/IUI, ovulation induction and/or intrauterine insemination; RD, risk difference; NC-infertile, naturally conceived infertile births that were conceived naturally by couples with a history of infertility; NC-fertile, naturally conceived fertile births that were conceived naturally by couples without a history of infertility. A closed circle indicates a statistically significant aRD; an open circle indicates a non-statistically significant aRD. Statistically significant results are indicated by coloured type.

In the propensity score-weighted cohort, compared to NC-fertile controls, ART-conceived infants had a higher risk of stillbirth (aRD 1.5 per 1000 births, 95% CI 0.4–2.7), perinatal mortality (aRD 1.8 per 1000 births, 95% CI 0.5–3.1), preterm birth (aRD 25.7 per 1000 births, 95% CI 21.3–30), admission to SCN/NICU (aRD 8.4 per 1000 births, 95% CI 1.2–15.6), and hospital admission <2 years of age (aRD 24.6 per 1000 births, 95% CI 17.2–32.0). However, when compared to NC-infertile controls, the risk for stillbirth (aRD 0.8 per 1000 births, 95% CI −0.4 to 2.1) and perinatal mortality (aRD 0.5 per 1000 births, 95% CI −1.0 to 1.9) were reduced by half or more, and the risks for preterm birth (aRD 9.5 per 1000 births, 95% CI 4.8–14.2), admission to SCN/NICU (−0.7 per 1000 births, 95% CI −8.0 to 6.6), and hospital admission <2 years of age (aRD 10.6 per 1000 births, 95% CI 2.5–18.7) were also notably reduced. The findings for OI/IUI-conceived controls were similar, except that a higher risk of SGA and lower risk of LGA were noted (Fig. 2).

In comparison with NC-fertile controls, NC-infertile controls had higher risks of preterm birth (aRD 8.5 per 1000 births, 95% CI 5.4–11.6), LGA (aRD 9.9 per 1000 births, 95% CI 5.8–14.0), and hospital admission <2 years of age (aRD 21.7 per 1000 births, 95% CI 15.7–27.7) (Supplementary Table S5), but a lower risk for SGA (aRD −19.1 per 1000 births, 95% CI −22.6 to −15.6).

### Subgroup analysis for ICSI and IVF

**Figure 3.**
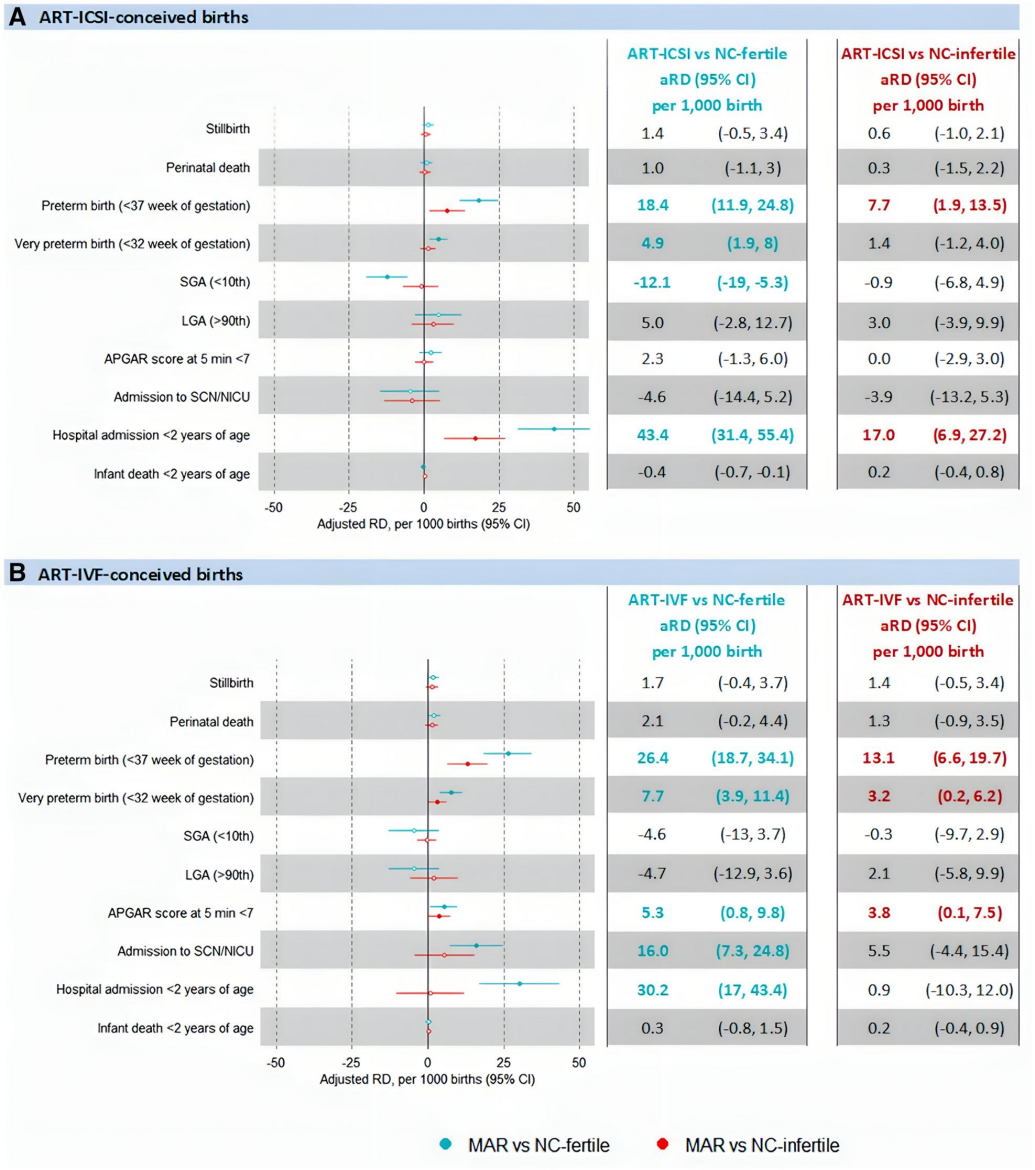
**Adjusted risk difference (aRD) for adverse perinatal outcomes for ART-ICSI and ART-IVF-conceived births compared to the two groups of naturally conceived (NC) controls, Singletons, NSW, 2009–2017 (N = 27 796).**  **A**) ART-ICSI-conceived births. **B**) ART-IVF-conceived births. RD, risk difference; NC-infertile, naturally conceived infertile births that were conceived naturally by couples with a history of infertility; NC-fertile, naturally conceived fertile births that were conceived naturally by couples without a history of infertility. A closed circle indicates a statistically significant aRD; an open circle indicates a non-statistically significant aRD. Statistically significant results are indicated by coloured type.

Infants conceived via ICSI (aRD 18.4 per 1000 births, 95% CI 11.9–24.8) and IVF (aRD 26.4 per 1000 births, 95% CI 18.7–34.1) had a higher incidence of preterm birth compared to NC-fertile controls, but this risk notably decreased when they were compared with NC-infertile controls (aRD 7.7 per 1000 births, 95% CI 1.9–13.5 for ICSI; 13.1 per 1000 births, 95% CI 6.6–19.7 for IVF). The likelihood of admission to SCN/NICU was higher in only IVF-conceived infants compared to NC-fertile controls (aRD 16.0 per 1000 births, 95% CI 7.3–24.8). This risk decreased substantially when compared to NC-infertile controls (aRD 5.5 per 1000 births, 95% CI −4.4 to 15.4). Hospital admission rates <2 years of age were higher in both ICSI (aRD 43.4 per 1000 births, 95% CI 31.4–55.4) and IVF (aRD 30.2 per 1000 births, 95% CI 17.0–43.4) -conceived infants. Similarly, this risk notably decreased when compared to NC-infertile controls (aRD 17.0 per 1000 births, 95% CI 6.9–27.2 for ICSI; 0.9 per 1000 births, 95% CI −10.3 to 12.0 for IVF) (Fig. 3).

### Subgroup analysis for fresh and frozen embryo transfer

**Figure 4.**
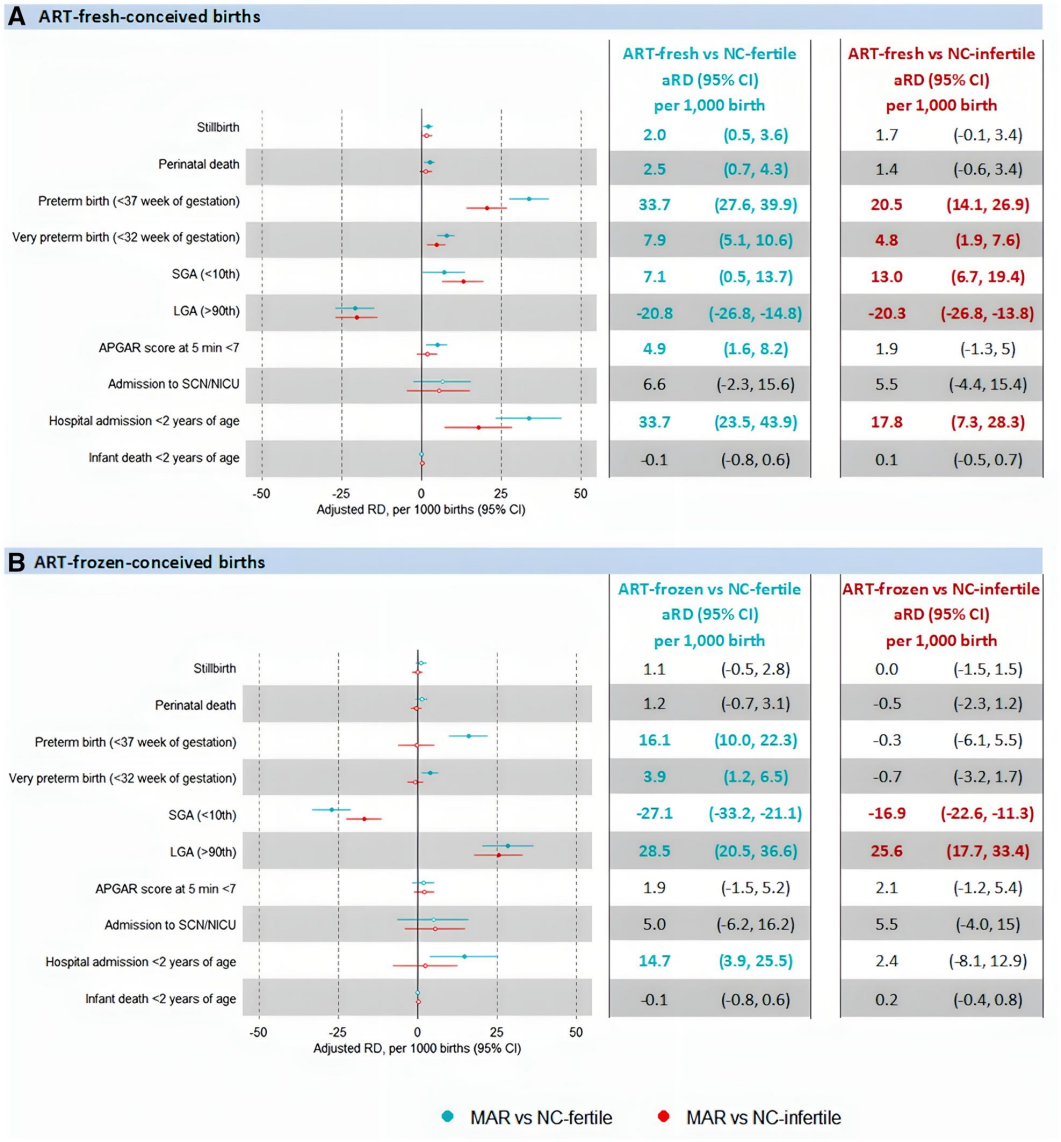
**Adjusted risk difference (aRD) for adverse perinatal outcomes for ART-Fresh and ART-Frozen-conceived births compared to the two naturally groups of conceived (NC) controls, singletons, NSW, 2009–2017 (N = 27 796). A**) ART-fresh-conceived births. **B**) ART-frozen-conceived births. MAR, medically assisted reproduction; RD, risk difference; ART-fresh, fresh embryo transfers; ART-frozen, frozen embryo transfers; NC-infertile, naturally conceived infertile births that were conceived naturally by couples with a history of infertility; NC-fertile, naturally conceived fertile births that were conceived naturally by couples without a history of infertility. A closed circle indicates a statistically significant aRD; an open circle indicates a non-statistically significant aRD. Statistically significant results are indicated by coloured type.

Compared to NC-fertile controls, ART-fresh embryo-conceived infants had a higher risk of preterm birth (aRD 33.7 per 1000 births, 95% CI 27.6–39.9) and hospital admission <2 years of age (aRD 33.7 per 1000 births, 95% CI 23.5–43.9). When compared to NC-infertile controls, the risk of preterm birth (aRD 20.5 per 1000 births, 95% CI 14.1–26.9) and hospital admission <2 years of age (aRD 17.8 per 1000 births, 95% CI 7.3–28.3) in ART-fresh-conceived infants was reduced. Infants conceived through fresh embryo transfer also had higher risks of stillbirth and perinatal mortality compared to NC-fertile controls. However, this risk was not evident in infants conceived using frozen embryo transfer. Frozen embryo transfer was associated with an increased risk of LGA (aRD 28.5 per 1000 births, 95% CI 20.5–36.6) and hospital admission <2 years of age (aRD 14.7 per 1000 births, 95% CI 3.9–25.5) when compared to NC-fertile controls. While the elevated risk of LGA persisted when compared to NC-infertile controls, the risk of hospital admission was significantly decreased (aRD 2.4 per 1000 births, 95% CI −8.1 to 12.9) (Fig. 4).

## Discussion

In this statewide cohort study, singletons conceived through ART or OI/IUI had higher risks of stillbirth, preterm birth, NICU admission, and infant hospitalization compared to naturally conceived infants without parental infertility. However, when compared to naturally conceived infants with parental infertility, these risks were notably reduced.

Assessing whether MAR increases risks of adverse perinatal and infant outcomes is imperative given the increasing global use of these technologies and concerns about their potential harm. An evidence-based approach is necessary to address legitimate concerns about MAR while ensuring access for those requiring these services. However, separating the individual contributions of MAR and infertility is challenging, as MAR is typically indicated by infertility and used as its treatment (Berntsen *et al.*, 2019).

Previous evidence consistently shows that singletons conceived through ART are at higher risk of adverse perinatal outcomes, including preterm birth, low birth weight, and SGA, compared to naturally conceived counterparts (Henningsen *et al.*, 2011; Pinborg *et al.*, 2013; Luke, 2017). These risks are likely influenced by both parental factors, such as advanced maternal age and underlying infertility, and ART-specific procedures, including ovarian stimulation, embryo culture, and the type of embryo transfer (Henningsen *et al.*, 2011; Pinborg *et al.*, 2013).

A sibling design that compares ART-conceived children to their biological siblings who were conceived naturally may assist in separating the effects of infertility and ART. Using this design, an earlier sibling study suggested that ART plays a role in the risk of low birth weight and preterm birth even when controlling for parental factors (Henningsen *et al.*, 2011). However, a later sibling design study reported that the increased risk of low birth weight and preterm birth in children conceived by ART is largely attributable to infertility but not the ART treatment (Goisis *et al.*, 2019). While the sibling design helps to control for shared parental factors that may be related to infertility, it only permits studying couples with milder forms of infertility who have a decreased but still reasonable chance of natural conception. Therefore, sibling studies could not represent all types of infertility and their associated risks. It is also essential to investigate the longer-term health outcomes of ART-conceived children, such as early childhood hospitalization, and to understand the impact of variations in ART techniques.

In our study, we included couples with all types of infertility and uniquely introduced an NC infertile control group to differentiate the effects of infertility from those of its treatment. We examined outcomes including Apgar scores at birth, admission to SCN/NICU, hospitalization, and mortality in early childhood. Furthermore, we analysed the effects of specific ART techniques, including ICSI versus IVF and fresh versus frozen embryo transfers, separately.

Previous research indicated that ICSI singletons had a lower risk of preterm birth than IVF singletons (Pinborg *et al.*, 2013), which may be because in ICSI cases, most of the women are reproductively healthy. We found that both IVF and ICSI were associated with an increased risk of preterm birth compared to the NC-infertile cohort, with the risk being notably higher for IVF. Notably, ICSI was associated with a higher risk of hospitalization in the early life of the infant compared to IVF, and this is only partially explained by infertility. We previously found that ICSI is an independent risk for major genitourinary abnormalities, particularly in couples without male infertility (Venetis *et al.*, 2023). These findings suggest that ICSI should be reserved only for couples with male infertility.

Previous research has consistently shown that while frozen embryo transfer reduces the risks of preterm birth and low birth weight compared to fresh embryo transfer, its association with LGA infants and macrosomia remains a concern (Wennerholm *et al.*, 2013; Luke *et al.*, 2017; Maheshwari *et al.*, 2018; Westvik-Johari *et al.*, 2021). Our findings align with these established associations regarding preterm birth and birth weight. Additionally, leveraging data linkage for extended follow-up, we identified that fresh embryo transfer was associated with an increased risk of hospital admission within the first 2 years of life, independent of underlying infertility.

A meta-analysis found comparable risks of perinatal mortality between fresh and frozen embryo transfers (Maheshwari *et al.*, 2018). More recently, a Scandinavian population-based cohort study found the risk of stillbirth was similar after fresh transfer and frozen transfer compared with singletons conceived without medical assistance, whereas neonatal mortality was high (Westvik-Johari *et al.*, 2023). In contrast, we observed an increased risk of stillbirth and perinatal death only with fresh embryo transfer. In our study population, the increased use of preimplantation genetic testing in frozen embryo transfer cycles may contribute to the observed differences between the fresh and frozen embryo transfers. Preimplantation genetic testing selects embryos without chromosomal abnormalities for transfer, which is more likely to result in healthier pregnancies. Its use in Australia increased from 1.7% in 2009 to 13.0% in 2017 (Assisted Reproductive Technology in Australia and New Zealand 2009, 2011; Assisted Reproductive Technology in Australia and New Zealand 2017, 2019).

We used the NSW Perinatal Data Collection (PDC) to identify all births and the incidence of most perinatal outcomes. The PDC captures comprehensive data on both home and hospital births in NSW regardless of the mode of conception. This dataset serves as the foundational cohort for births in our study. Couples who become parents after MAR and NC-infertile controls may be more likely to exhibit help-seeking behaviours and have more frequent interactions with the healthcare system than the NC-fertile controls, potentially leading to improved perinatal outcomes. However, evidence from low-risk pregnancies suggests that more frequent prenatal visits do not necessarily improve neonatal outcomes (Carter *et al.*, 2016). Moreover, naturally conceived pregnancies in our population should have similar healthcare as those conceived by MAR. In 2022, only 0.2% of Australian mothers had no antenatal visits, and over 90% attended at least five antenatal care appointments (Australian Institute of Health and Welfare, 2024a). Thus, differences in help-seeking behaviour are unlikely to affect the findings of this study.

Our study has several limitations. First, the observational nature of the study, coupled with the use of administrative data, may carry a risk of misclassification or unmeasured confounding bias. In particular, our study cannot assess whether couples changed partners during the study period. This may have led to a small degree of misclassification, particularly in the NC-infertile cohort, where a female partner might have previously undergone ART or IUI due to a former partner’s male infertility or biological incompatibility. Second, we focused on singleton births because the risk profile for multiple births differs significantly from that of singletons, warranting separate in-depth investigations. Third, although we built a large cohort, the precision of estimates for some very rare outcomes was low when separating ART variations. Fourth, parents with a history of infertility who achieved natural pregnancy likely had less severe underlying conditions than those who can only achieve pregnancy via MAR, leading potentially to an underestimation of the contribution of infertility. However, had these parents had more severe infertility, we expect an even smaller contribution of MAR to adverse perinatal outcomes, strengthening the conclusion. Fifth, the study cohort only included children born at or after 20 weeks’ gestation and could not account for early pregnancy loss in the analysis. Lastly, socio-economic confounding remains a possibility, however, the residential SIEFA code used in our analysis takes account of income, education, employment, occupation, housing, and family structure. Also, information on BMI was not available.

This study highlights the need for enhanced obstetric and neonatal surveillance in MAR pregnancies. While the findings of this study provide some reassurance that the increased risk is not largely related to MAR, they also show that ART technologies may partially contribute to some of these risks, emphasizing the importance of reserving ART where ART treatment is indicated.

In conclusion, while singletons conceived through MAR have an elevated risk of adverse perinatal outcomes, these risks are mainly associated with underlying parental infertility that led to the use of MAR technologies. ICSI is linked to an increased risk of hospital admission in early life, while IVF is associated with a higher likelihood of preterm birth. Fresh embryo transfer poses greater concerns than frozen embryo transfer regarding preterm birth, SGA, and hospital admission in early life.

## Supplementary Material

hoaf020_Supplementary_Data

## Data Availability

Data sharing agreements and ethics approvals of this study prohibit the study team from making the dataset publicly available.
