# Supplementary material for: Perinatal risks associated with infertility and medically assisted reproduction: a population-based cohort study
Source: Hum Reprod Open. 2025 May 8;2025(2):hoaf020. doi: 10.1093/hropen/hoaf020 (PMC12124915; doi:10.1093/hropen/hoaf020)

**Perinatal risks associated with infertility and medically assisted reproduction: a population-based cohort study**

Stephanie K.Y. Choi, Wentao Li, Christos Venetis, William Ledger, Kei Lui, Katie Harris, Robert J Norman, Louisa R Jorm , Georgina M Chambers

**Supplementary Data**

Supplementary Table S1. Registries for data-linkage in the singleton’s cohort, NSW, 2009-2017

Supplementary Table S2. Criteria for identifying NC-infertile births

Supplementary Table S3. Baseline characteristics for ART-conceived births, Singletons, NSW, 2009-2017 (N=27,796)

Supplementary Table S4. Crude incidence of adverse perinatal outcomes in singletons by mode of conception, NSW, 2009-2017

Supplementary Table S5. Adjusted risk difference (aRD) for adverse perinatal outcomes for naturally conceived infertile control compared to the naturally conceived fertile control, Singletons, NSW, 2009-2017 (N=783,269)

Supplementary Figure S1. Balance analysis for covariates comparing MAR-conceived singletons to naturally conceived **fertile** controls before and after propensity-score weighting

Supplementary Figure S2. Balance analysis for covariates comparing MAR-conceived singletons to naturally conceived **infertile** controls before and after propensity-score weighting

Supplementary Figure S3. Crude incidence of adverse perinatal outcomes, by mode of conception, singletons, NSW, 2009-2017 (N=824,639)

| Registry / data set | Description | Information source in the study cohort |
| --- | --- | --- |
| Australia and New Zealand Assisted Reproduction Database (ANZARD) | A clinical quality registry that systematically collects data on all assisted reproductive technology treatment cycles performed in fertility clinics across Australia and New Zealand. | Exposure: Assisted reproductive technology treatment (including fertilization method and embryo transfer type) and intrauterine insemination. |
| Australian Medicare Benefits Schedule (MBS) | A listing of medical services subsidized by the Australian government under the Medicare program, includes consultations, procedures, diagnostic tests, and therapeutic treatments. | Exposure: Assisted reproductive technology service; intrauterine insemination service |
| Australian Pharmaceutical Benefits Scheme (PBS) | A listing of subsidized prescription medicines that have been approved for listing by the Australian government. | Exposure: Medicines for ovulation induction, intrauterine insemination, and assisted reproductive technology treatment  Covariates: maternal pre-existing health conditions and pregnancy-related health conditions |
| New South Wales Perinatal Data Collection (PDC) | A statewide surveillance system that monitors patterns of pregnancy care, services and pregnancy. | Outcome: Stillbirth, perinatal death, preterm birth, small-for-gestational age, large-for-gestational age, APGAR score, and admission to NICU  Covariates: maternal age, other parent’s age, baby’s sex, smoking in pregnancy, parity, Australian-born, place of residing, year of birth, maternal pre-existing health conditions, and pregnancy-related health conditions |
| New South Wales Registry of Births, Deaths & Marriages (RBDM) | A government agency that registers and maintains official records of all births, deaths, marriages, and changes of name that occur in the state. | Outcome: Perinatal death  Covariates: other partner’s age, baby’s sex, parity, and pregnancy-related health conditions |
| New South Wales Cause of Death Unit Record File (COD-URF) | An administrative dataset maintained by the Ministry of Health that contains detailed information on all deaths that have occurred in the state. | Outcome: Perinatal death |
| New South Wales Admitted Patient Data Collection | An administrative data collection that captures information on all inpatient hospitalizations and day-only procedures that occur in public and private hospitals across the state. | Outcome: Hospital admission for offspring.  Covariates: maternal pre-existing health conditions and pregnancy-related health conditions |

**Supplementary Table S1. Registries for data-linkage in the singleton’s cohort, NSW, 2009-2017**

**Supplementary Table S2. Criteria applied to the naturally conceived births cohort to identify NC-infertile births.**

**More details of IVF-specific and OI-specific fertility medicine assisted reproductive technology-related or tubal patency testing procedure records can be found in Chambers et al (2021).**

| **Criteria** | **Data Sources** | **Criteria** |
| --- | --- | --- |
| **1** | **PBS** | At least one dispensing of IVF-specific and OI-specific fertility medicine dispensing between the earliest available PBS records and 3 months prior to the date of conception |
| **2** | **MBS** | At least one record of assisted reproductive technology-related or tubal patency testing procedure between the date of the earliest available MBS record and three months prior to the date of conception |
| **3** | **ANZARD** | At least one non-oocyte donation ANZARD record between the date of the earliest available ANZARD record and 1 month prior to the date of conception |
| **4** | **PBS and ANZARD** | There was an IVF-specific medicine record that appeared during 3 months prior to the date of conception AND within 60 days following this fertile medicine record, there was an embryo transfer happened without a clinical pregnancy outcome. |

ANZARD = Australia and New Zealand Assisted Reproduction Database; MBS = Medicare Benefits Schedule; PBS = Pharmaceutical Benefits Scheme; IVF =In vitro fertilization; OI=ovulation induction

**Supplementary Table S3. Baseline characteristics for ART-conceived births, Singleton, NSW, 2009-2017 (N=27,796)**

| **Baseline characteristics** | **ART ICSI vs IVF** | | **ART fresh vs frozen** | |
| --- | --- | --- | --- | --- |
|  | **ICSI-conceived**  **(N=14,466)** | **IVF-conceived**  **(N=13,330)** | **Fresh-conceived**  **(N=15,436)** | **Frozen-conceived**  **(N=12,360)** |
| **Maternal age, at delivery** |  |  |  |  |
| < 30 years | 1,662 (11.5%) | 1,225 (9.2%) | 1,842 (11.9%) | 1,045 (8.5%) |
| 30-34 years | 4,902 (33.9%) | 4,554 (34.2%) | 5,515 (35.7%) | 3,941 (31.9%) |
| 35-39 years | 5,757 (39.8%) | 5,472 (41.1%) | 6,019 (39.0%) | 5,210 (42.2%) |
| ≥40 years | 2,145 (14.8%) | 2,079 (15.6%) | 2,060 (13.4%) | 2,164 (17.5%) |
| **Other parent’s age, at delivery** |  |  |  |  |
| < 30 years | 789 (5.5%) | 632 (4.7%) | 949 (6.2%) | 472 (3.8%) |
| 30-34 years | 3,374 (23.3%) | 3,298 (24.7%) | 4,027 (26.1%) | 2,645 (21.4%) |
| 35-39 years | 4,657 (32.2%) | 4,909 (36.8%) | 5,216 (33.8%) | 4,350 (35.2%) |
| ≥40 years | 4,945 (34.2%) | 3,747 (28.1%) | 4,484 (29.1%) | 4,208 (34.1%) |
| **Baby’s sex, male** | 7,449 (51.5%) | 7,315 (54.9%) | 8,355 (54.1%) | 6,409 (51.9%) |
| **Smoking during pregnancy** | 215 (1.5%) | 199 (1.5%) | 262 (1.7%) | 152 (1.2%) |
| **Nulliparous** | 9,266 (64.5%) | 8,384 (62.9%) | 11,309 (73.3%) | 7,278 (53.8%) |
| **Australia-born** | 9,906 (68.5%) | 9,094 (62.8%) | 10,209 (66.1%) | 8,081 (65.4%) |
| **Residing in most socially disadvantaged areas** | 4,150 (28.7%) | 3,860 (29.0%) | 4,619 (29.9%) | 3,391 (27.4%) |
| **Residing in major cities** | 12,688 (87.7%) | 11,829 (88.7%) | 13,560 (87.9%) | 10,957 (88.6%) |
| **Year of birth** |  |  |  |  |
| 2009 | 1,115 (7.7%) | 1,439 (10.8%) | 1,754 (11.4%) | 800 (6.5%) |
| 2010 | 1,421 (9.8%) | 1,301 (9.8%) | 1,836 (11.9%) | 886 (7.2%) |
| 2011 | 1,507 (10.4%) | 1,114 (8.4%) | 1,676 (10.9%) | 945 (7.7%) |
| 2012 | 1,721 (11.9%) | 1,267 (9.5%) | 1,912 (12.4%) | 1,076 (8.7%) |
| 2013 | 1,754 (12.1%) | 1,380 (10.4%) | 1,776 (11.5%) | 1,358 (11.0%) |
| 2014 | 1,780 (12.3%) | 1,489 (11.2%) | 1,744 (11.3%) | 1,525 (12.3%) |
| 2015 | 1,767 (12.2%) | 1,531 (11.5%) | 1,624 (10.5%) | 1,674 (13.5%) |
| 2016 | 1,843 (12.7%) | 1,771 (13.3%) | 1,649 (10.7%) | 1,965 (15.9%) |
| 2017 | 1,558 (10.8%) | 2,038 (15.3%) | 1,465 (9.5%) | 2,131 (17.2%) |
| **Pre-existing comorbidities ^a^** |  |  |  |  |
| Diabetes | 889 (6.2%) | 1,001 (7.5%) | 1,020 (6.6%) | 870 (7.0%) |
| Hypertension | 139 (1.0%) | 118 (0.9%) | 132 (0.9%) | 125 (1.0%) |
| Alcohol and drug dependence | 29 (0.2%) | 18 (0.1%) | 28 (0.2%) | 19 (0.2%) |
| Mental disorder | 1,227 (8.5%) | 1,025 (7.7%) | 1,158 (7.5%) | 1,094 (8.9%) |
| Chronic airway disease | 1,243 (8.6%) | 1,064 (8.0%) | 1,275 (8.3%) | 1,032 (8.4%) |
| Cancer | 56 (0.4%) | 49 (0.4%) | 39 (0.3%) | 66 (0.5%) |
| Cardiovascular disease | 254 (1.8%) | 196 (1.5%) | 225 (1.5%) | 225 (1.9%) |
| Thyroid | 782 (5.4%) | 788 (5.9%) | 738 (4.8%) | 832 (6.7%) |
| Gastro-oesophageal reflux disease | 1,017 (7.0%) | 854 (6.4%) | 967 (6.3%) | 904 (7.3%) |
| Epilepsy | 84 (0.6%) | 76 (0.6%) | 95 (0.6%) | 65 (0.5%) |
| Anaemia and coagulation | 1,368 (9.5%) | 1,016 (7.6%) | 920 (6.0%) | 1,464 (11.8%) |
| Inflammation/pain | 2,336 (16.2%) | 2,337 (17.5%) | 2,240 (14.5%) | 2,433 (19.7%) |
| Steroid responsive disease | 894 (6.2%) | 700 (5.3%) | 667 (4.3%) | 927 (7.5%) |
| Irritable bowel disease | 100 (0.7%) | 127 (1.0%) | 118 (0.8%) | 109 (0.9%) |
| Liver disease | 52 (0.4%) | 26 (0.2%) | 33 (0.2%) | 45 (0.4%) |
| Rheumatic disease | 969 (6.7%) | 903 (6.8%) | 919 (6.0%) | 953 (7.7%) |
| Obesity | 51 (0.4%) | 35 (0.1%) | 56 (0.4%) | 30 (0.2%) |
| **Pregnancy-related comorbidities** |  |  |  |  |
| Cervical incompetence | 155 (1.1%) | 144 (1.1%) | 154 (1.0%) | 145 (1.2%) |
| Fibroid uterus | 67 (0.5%) | 57 (0.4%) | 71 (0.5%) | 53 (0.4%) |
| Congenital uterine anomalies | 13 (0.1%) | 15 (0.1%) | 11 (0.1%) | 17 (0.1%) |
| Cardiovascular disease | 155 (1.1%) | 139 (1.0%) | 152 (1.0%) | 142 (1.2%) |
| Asthma during pregnancy | 207 (1.4%) | 205 (1.5%) | 211 (1.4%) | 201 (1.6%) |
| Alcohol and drug dependence | 5 (0.03%) | 5 (0.03%) | 7 (0.05%) | <5 |
| Renal disease | 105 (0.8%) | 112 (0.8%) | 115 (0.8%) | 102 (0.8%) |
| History of preterm births | 146 (1.0%) | 136 (1.0%) | 89 (0.6%) | 193 (1.6%) |
| History of miscarriage | 1,197 (8.3%) | 1,115 (8.4%) | 1,050 (6.8%) | 1,262 (10.2%) |
| History of antepartum haemorrhage | 89 (0.6%) | 67 (0.5%) | 50 (0.3%) | 106 (0.9%) |
| History of prelabour-preterm rupture of membranes | 286 (2.0%) | 263 (2.0%) | 157 (1.0%) | 392 (3.2%) |

ART= Assisted reproductive technology; ICSI= intracytoplasmic sperm injection; IVF= In vitro fertilization

^a^ Pre-existing comorbidities were determined two years before the date of conception.

**Supplementary Table S4. Crude incidence of adverse perinatal outcomes in singletons by mode of conception, NSW, 2009-2017**

| **Adverse perinatal outcome** | **N** | **incidence (%)** | **95% LCI** | **95% UCI** |
| --- | --- | --- | --- | --- |
| **ART (N=27,796)** |  |  |  |  |
| Any adverse perinatal outcome | 10,538 | 37.34 | 37.34 | 38.48 |
| Perinatal death | 270 | 0.97 | 0.86 | 1.09 |
| Stillbirth | 186 | 0.67 | 0.58 | 0.77 |
| SGA, <10th | 2,476 | 8.91 | 8.58 | 9.25 |
| LGA, >90th | 2,695 | 9.70 | 9.35 | 10.05 |
| Preterm | 2,420 | 8.71 | 8.38 | 9.04 |
| Very preterm | 496 | 1.78 | 1.64 | 1.95 |
| APGAR score at 5 min, <7 | 679 | 2.44 | 2.27 | 2.63 |
| Admission to SCN/NICU | 2,930 | 14.23 | 13.76 | 14.72 |
| Hospital admission, <2 yr | 8,137 | 28.27 | 28.74 | 29.81 |
| Infant death, 1 mo-< 2 yr | 19 | 0.07 | 0.04 | 0.11 |
| **OI/IUI (N=13,574)** |  |  |  |  |
| Any adverse perinatal outcome | 5,754 | 42.39 | 41.56 | 43.22 |
| Perinatal death | 124 | 0.91 | 0.76 | 1.08 |
| Stillbirth | 79 | 0.58 | 0.47 | 0.73 |
| SGA, <10th | 1,509 | 11.12 | 10.61 | 11.66 |
| LGA, >90th | 1,193 | 8.79 | 8.32 | 9.28 |
| Preterm | 1,015 | 7.48 | 7.05 | 7.93 |
| Very preterm | 200 | 1.47 | 1.28 | 1.69 |
| APGAR score at 5 min, <7 | 316 | 2.33 | 2.09 | 2.6 |
| Admission to SCN/NICU | 2,146 | 15.81 | 15.14 | 16.5 |
| Hospital admission, <2 yr | 4,067 | 29.96 | 29.2 | 30.74 |
| Infant death, 1 mo-< 2 yr | 14 | 0.1 | 0.06 | 0.16 |
| **NC-infertile (N=36,521)** | |  |  |  |
| Any adverse perinatal outcome | 14,115 | 38.65 | 38.15 | 39.15 |
| Perinatal death | 310 | 0.85 | 0.76 | 0.95 |
| Stillbirth | 212 | 0.58 | 0.51 | 0.66 |
| SGA, <10th | 2,867 | 7.85 | 7.58 | 8.13 |
| LGA, >90th | 3,992 | 10.93 | 10.61 | 11.25 |
| Preterm | 2,615 | 7.16 | 6.9 | 7.43 |
| Very preterm | 504 | 1.38 | 1.26 | 1.5 |
| APGAR score at 5 min, <7 | 734 | 2.01 | 1.87 | 2.16 |
| Admission to SCN/NICU | 4,978 | 13.63 | 13.23 | 14.04 |
| Hospital admission, <2 yr | 10,463 | 28.65 | 28.19 | 29.12 |
| Infant death, 1 mo-< 2 yr | 22 | 0.06 | 0.04 | 0.09 |
| **NC-fertile (N=747,018)** | |  |  |  |
| Any adverse perinatal outcome | 279,684 | 37.44 | 37.33 | 37.54 |
| Perinatal death | 5,453 | 0.73 | 0.72 | 0.75 |
| Stillbirth | 3,810 | 0.51 | 0.5 | 0.53 |
| SGA, <10th | 72,013 | 9.64 | 9.57 | 9.71 |
| LGA, >90th | 72,386 | 9.69 | 9.63 | 9.76 |
| Preterm | 43,028 | 5.76 | 5.71 | 5.82 |
| Very preterm | 7,844 | 1.05 | 1.02 | 1.07 |
| APGAR score at 5 min, <7 | 15,314 | 2.05 | 2.02 | 2.08 |
| Admission to SCN/NICU | 99,055 | 13.26 | 13.17 | 13.35 |
| Hospital admission, <2 yr | 195,196 | 26.13 | 26.03 | 26.23 |
| Infant death, 1 mo-< 2 yr | 672 | 0.09 | 0.08 | 0.1 |
| **ART-ICSI (N=14,466)** |  |  |  |  |
| Any adverse perinatal outcome | 5,670 | 39.20 | 38.40 | 39.99 |
| Perinatal death | 136 | 0.94 | 0.80 | 1.11 |
| Stillbirth | 92 | 0.64 | 0.52 | 0.78 |
| SGA, <10th | 1,236 | 8.54 | 8.10 | 9.01 |
| LGA, >90th | 1,493 | 10.32 | 9.84 | 10.83 |
| Preterm | 1,221 | 8.44 | 8.00 | 8.90 |
| Very preterm | 246 | 1.70 | 1.50 | 1.92 |
| APGAR score at 5 min, <7 | 337 | 2.33 | 2.10 | 2.59 |
| Admission to SCN/NICU | 1,521 | 13.75 | 13.12 | 14.40 |
| Hospital admission, <2 yr | 4,362 | 30.15 | 29.41 | 30.90 |
| Infant death, 1 mo-< 2 yr | 10 | 0.07 | 0.04 | 0.13 |
| **ART-IVF (N=13,330)** |  |  |  |  |
| Any adverse perinatal outcome | 4,868 | 36.52 | 35.71 | 37.34 |
| Perinatal death | 134 | 1.01 | 0.85 | 1.19 |
| Stillbirth | 94 | 0.71 | 0.58 | 0.86 |
| SGA, <10th | 1,240 | 9.30 | 8.82 | 9.81 |
| LGA, >90th | 1,202 | 9.02 | 8.54 | 9.52 |
| Preterm | 1,199 | 8.99 | 8.52 | 9.49 |
| Very preterm | 250 | 1.88 | 1.66 | 2.12 |
| APGAR score at 5 min, <7 | 342 | 2.57 | 2.31 | 2.85 |
| Admission to SCN/NICU | 1,409 | 14.80 | 14.10 | 15.53 |
| Hospital admission, <2 yr | 3,775 | 28.32 | 27.56 | 29.09 |
| Infant death, 1 mo-< 2 yr | 9 | 0.07 | 0.04 | 0.13 |
| **ART-fresh embryo transfer (N=15,436)** | |  |  |  |
| Any adverse perinatal outcome | 6,337 | 41.05 | 40.28 | 41.83 |
| Perinatal death | 172 | 1.11 | 0.96 | 1.29 |
| Stillbirth | 120 | 0.78 | 0.65 | 0.93 |
| SGA, <10th | 1,630 | 10.56 | 10.08 | 11.05 |
| LGA, >90th | 1,189 | 7.70 | 7.29 | 8.13 |
| Preterm | 1,500 | 9.72 | 9.26 | 10.19 |
| Very preterm | 318 | 2.06 | 1.85 | 2.30 |
| APGAR score at 5 min, <7 | 397 | 2.57 | 2.33 | 2.83 |
| Admission to SCN/NICU | 1,824 | 14.80 | 14.19 | 15.44 |
| Hospital admission, <2 yr | 4,634 | 30.02 | 29.30 | 30.75 |
| Infant death, 1 mo-< 2 yr | 10 | 0.06 | 0.03 | 0.12 |
| **ART-frozen embryo transfer (N=12,360)** | |  |  |  |
| Any adverse perinatal outcome | 4,201 | 33.99 | 33.16 | 34.83 |
| Perinatal death | 98 | 0.79 | 0.65 | 0.97 |
| Stillbirth | 66 | 0.53 | 0.42 | 0.68 |
| SGA, <10th | 846 | 6.84 | 6.41 | 7.30 |
| LGA, >90th | 1,506 | 12.18 | 11.62 | 12.77 |
| Preterm | 920 | 7.44 | 6.99 | 7.92 |
| Very preterm | 178 | 1.44 | 1.24 | 1.67 |
| APGAR score at 5 min, <7 | 282 | 2.28 | 2.03 | 2.56 |
| Admission to SCN/NICU | 1,106 | 13.38 | 12.67 | 14.13 |
| Hospital admission, <2 yr | 3,503 | 28.34 | 27.55 | 29.14 |
| Infant death, 1 mo-< 2 yr | 9 | 0.07 | 0.04 | 0.14 |

ART= Assisted reproductive technology; ICSI= intracytoplasmic sperm injection; IVF= In vitro fertilization; SGA=small-for-gestational-age; LGA=large-for-gestational-age; NICU=neonatal intensive care unit; LCI=lower confidence interval; UCI=upper confidence interval

**Supplementary Table S5. Adjusted risk difference (aRD) for adverse perinatal outcomes for naturally conceived infertile control compared to the naturally conceived fertile control, Singletons, NSW, 2009-2017 (N=783,269)**

|  | **NC-infertile vs NC-fertile** | |
| --- | --- | --- |
|  | **aRD (95% CI)** | |
|  | **per 1,000 birth** | |
| Stillbirth | 0.5 | (-0.3, 1.4) |
| Perinatal death | 0.8 | (-0.2, 1.9) |
| Preterm birth (<37 week of gestation) | **8.5** | **(5.4, 11.6)** |
| Very preterm birth (<32 week of gestation) | **2.7** | **(1.3, 4.1)** |
| SGA (<10th) | **-19.1** | **(-22.6, -15.6)** |
| LGA (>90th) | **9.9** | **(5.8, 14.0)** |
| APGAR score at 5 min < 7 | -1.3 | (-3.0, 0.4) |
| Admission to SCN/NICU | 3.2 | (-1.9, 8.4) |
| Hospital admission < 2 years of age | **21.7** | **(15.7, 27.7)** |
| Infant death < 2 years of age | **-0.4** | **(-0.7, -0.2)** |

RD = risk difference;; ART-frozen = frozen embryo transfers; CI = confidence intervals; NC-infertile=Naturally conceived infertile births that were conceived naturally by mothers with a history of infertility; NC-fertile=Naturally conceived fertile births that were conceived naturally by mothers without a history of infertility

**Supplementary Figure S1. Balance analysis for covariates comparing MAR-conceived singletons to naturally conceived fertile controls before and after propensity-score weighting.**

ART = assisted reproductive technology; OI/IUI = ovulation induction and/or Intrauterine insemination; ICSI = intracytoplasmic sperm injection; IVF = in vitro fertilization; NC-infertile = naturally conceived infertile births that were conceived naturally by mothers with a history of infertility; NC-fertile= naturally-conceived fertile births that were conceived naturally by mothers without a history of infertility

A standardized difference of each covariate with a value of greater than 0.1 indicates a non-negligible difference between MAR-conceived birth and each of the two NC references

**Supplementary Figure S2. Balance analysis for covariates comparing MAR-conceived singletons to naturally conceived infertile controls before and after propensity-score weighting**

ART= assisted reproductive technology; OI/IUI = ovulation induction and/or Intrauterine insemination; ICSI= intracytoplasmic sperm injection; IVF= in vitro fertilization; NC-infertile = naturally-conceived infertile births that were conceived naturally by mothers with a history of infertility; NC-fertile = naturally-conceived fertile births that were conceived naturally by mothers without a history of infertility

A standardized difference of each covariate with a value of greater than 0.1 indicates a non-negligible difference between MAR-conceived birth and each of the two NC references.

**Supplementary Figure S3. Crude incidence of adverse perinatal outcomes, by mode of conception, singletons, NSW, 2009-2017 (N=824,639)**

ART= assisted reproductive technology; OI/IUI = ovulation induction and/or Intrauterine insemination; ICSI = intracytoplasmic sperm injection; IVF = in vitro fertilization

In each subplot, we stratified ART by ICSI and IVF; and by fresh and frozen embryo transfer. The lighter colour bars in each subplot indicate that these ART subgroups are not mutually exclusive


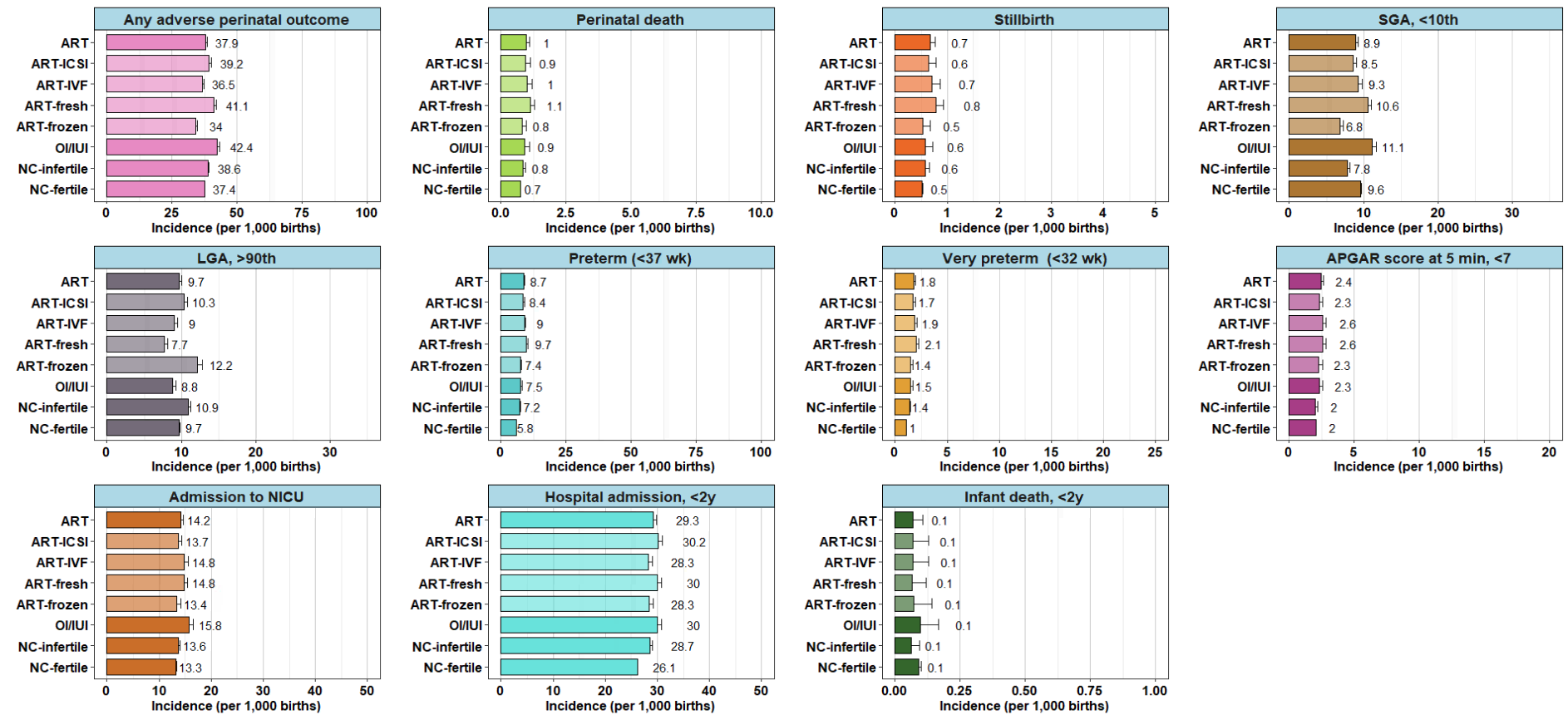

Supplement: hoaf020_Supplementary_Data [file hoaf020_supplementary_data.docx]
